# *Listeria monocytogenes* Contamination Characteristics in Two Ready-to-Eat Meat Plants From 2019 to 2020 in Shanghai

**DOI:** 10.3389/fmicb.2021.729114

**Published:** 2021-08-26

**Authors:** Hongzhi Zhang, Jing Wang, Zhaoyu Chang, Xin Liu, Weijie Chen, Ying Yu, Xiaoguang Wang, Qingli Dong, Yulong Ye, Xi Zhang

**Affiliations:** ^1^Shanghai Municipal Center for Disease Control and Prevention, Shanghai, China; ^2^The Minhang District Center for Disease Control and Prevention, Shanghai, China; ^3^Institute of Food Quality and Safety, University of Shanghai for Science and Technology, Shanghai, China; ^4^The Jinshan District Center for Disease Control and Prevention, Shanghai, China

**Keywords:** *Listeria monocytogenes*, ready-to-eat food processing plant, PFGE, WGS, cgMLST, SNP

## Abstract

*Listeria monocytogenes* is a ubiquitous foodborne pathogen that causes listeriosis and is mostly linked to consumption of ready-to-eat (RTE) foods. Lack of hygiene in food processing environments may be a primary reason for contamination by *L. monocytogenes* isolates. In this study, *L. monocytogenes* strains isolated from two RTE meat processing plants in the Shanghai municipality, China, were characterized during 2019–2020 using pulsed-field gel electrophoresis and whole-genome sequencing. Results showed that 29 samples (12.2%) out of 239 were positive for *L. monocytogenes*, with 21 (18.9%) and 8 (6.25%) isolates from plants A and B, respectively. The packaging room at plant A had the most contamination (14, 48.3%; *p* < 0.05), with a peak occurrence of 76.5% in processing environments. Nineteen *L. monocytogenes* isolates belonging to the pulsotype (PT) 7 group were indistinguishable (≥ 95.7%). Furthermore, core-genome multiple loci sequencing typing identified up to nine allelic differences, and the closet pairwise differences among these ST5 isolates included 0–16 small nucleotide polymorphisms. Therefore, *L. monocytogenes* likely persisted at plant A during 2019–2020 with ongoing clone transmission. In contrast, no *L. monocytogenes* isolates were identified from processing environments at plant B. Most *L. monocytogenes* isolates were sampled from raw materials (62.5%). Several isolates (ST378, ST8, and ST120) were detected only once in 2020 and were considered as transient isolates. However, three ST121 isolates with the same PT (PT2) were detected in 2020 and should be noted for their stronger survival ability in harsh environments. These results suggest that continuous monitoring, stringent surveillance, and source tracking are crucial to guaranteeing food safety in RTE food plants.

## Introduction

*Listeria monocytogenes* is a ubiquitous foodborne pathogen that causes listeriosis and is frequently linked to consumption of ready-to-eat (RTE) foods, such as fresh produce, soft cheese, and especially RTE meat products (Fagerlund et al., 2020). RTE meat products have caused several outbreaks of listeriosis, including the largest outbreak that occurred in South Africa (Smith et al., 2019; Gilmour et al., 2020). Furthermore, listeriosis remains a significant public health concern because of the high case fatality and mortality (Thomas et al., 2015).

RTE foods are intended to be consumed without further cooking or other processing, thus requiring the elimination or reduction of microorganisms of concern to acceptable levels. The microbiological safety of RTE foods relies on the adoption by manufacturers and processors of a number of measures aimed at preventing food contamination (FAO., and WHO., 2019). Contamination by *L. monocytogenes* is a major characteristic at food processing plants because bacterium can exist in harsh environments, such as low pH and high salt concentrations, and can grow and persist at low temperatures (Ferreira et al., 2014). The epidemiological investigation of meat-related outbreaks has revealed substantial deficiencies in hygiene in food processing environments (Smith et al., 2019). In a previous study, the contamination of *L. monocytogenes* in an RTE food processing plant suggested that clone transmission may occur in the food processing environments in Shanghai (Zhang et al., 2021) and thus pose a potential hazard to food safety. Therefore, the constant risk of *L. monocytogenes* transmission to consumers remains a central challenge to the food processing plants (Almeida et al., 2013; Muhterem-Uyar et al., 2015).

The multilocus sequence typing (MLST) method has been used to type *L. monocytogenes* into many different sequence types (STs). Epidemiological investigations have identified that several STs are predominantly found in clinical patients, whereas other STs have been shown to be particularly abundant in food or food processing environments (Maury et al., 2016). In China, ST87 and ST8 were the most STs isolated from patients (Li et al., 2019), whereas ST9 was the most common ST isolated from foods (Wang et al., 2012; Zhang et al., 2020). ST5 and ST121 isolates have been reported to be predominantly found in food processing environments (Muhterem-Uyar et al., 2015; Naditz et al., 2019; Zhang et al., 2021). Therefore, MLST of *L. monocytogenes* can help establish links between isolates from different sources and provide information regarding the source of contamination (Chen et al., 2013).

Pulsed-field gel electrophoresis (PFGE) has been used to characterize clusters of *L. monocytogenes* isolates and can even identify persistence (Buchanan et al., 2017; Li et al., 2019). Persistent strains are defined as those with an indistinguishable PFGE signature (Buchanan et al., 2017). Whole-genome sequencing (WGS) is a powerful tool for analyzing molecular characteristics of *L. monocytogenes* together with the development of core-genome MLST (cgMLST) and single nucleotide polymorphisms (SNPs) that have been used to investigate listeriosis outbreaks and offer various advantages over PFGE (Chen et al., 2016; Jackson et al., 2016). WGS has recently been used to determine the contamination and/or colonization route of pathogens within food processing environments (Zhen et al., 2017). Tracking the primary contamination route of *L. monocytogenes* in RTE meat processing plants is essential as this could help guide food business operators to remove or reduce resident *L. monocytogenes*. In this study, we investigated the contamination of *L. monocytogenes* in two RTE plants in Shanghai. The study aimed to (1) describe the occurrence of *L. monocytogenes* in different areas, such as thawing, trimming, boiling, cooled, packaging, and sterilizing rooms, at two RTE meat plants in Shanghai; (2) trace the relevant sources of contamination for RTE meat products using PFGE and WGS and clarify the transmission of *L. monocytogenes* at RTE meat plants; and (3) identify the molecular characteristics of persistent isolates in RTE meat plants in relation to their ST, serogroup, and virulence profile.

## Materials and Methods

### RTE Meat Product Processing

*L. monocytogenes* contamination was investigated in processing environments and products, and the transmission route tracked at two RTE meat processing plants in Shanghai. Plant A and plant B are well-established famous companies that produce RTE meat products in Shanghai. There is no connection between the two RTE plants. The processing procedure of RTE meat products at these two plants are similar, and the meat products were processed as follows: first, frozen meat was bought from trade companies as raw material. Second, the meat was water thawed in a thawing room, and cut and trimmed in the trimming room; following which, the meat was pickled in a pickling liquid with accessory materials including oil, salt, sauce, vinegar, and spices in pickling rooms. Third, the pickled meat products were boiled to produce intermediate products in boiling rooms. After being cooled in the cooling room, these intermediate products were eventually processed by activities such as weighing and cutting into shapes to create products in packaging rooms. Finally, these products were sterilized in a sterilization room to create end products for consumers.

### Sampling

A total of 239 samples were collected during one visit every year at the two RTE meat plants from 2019 to 2020 in Shanghai (Table 1). A total of 113 samples were collected at plant A during 2019 and 2020, including 16 raw materials, 4 accessory materials, 6 process water samples, 32 intermediate products, 42 processing environments and facilities samples, 6 products, and 3 end products in seven areas (thawing, trimming, pickling, boiling, cooled, packaging, and sterilizing rooms). A total of 128 samples were collected at plant B, including 35 raw materials, 6 process water samples, 17 intermediate products, 3 accessory materials, 55 processing environments and facilities samples, 3 products, and 9 end products from the same seven areas as described for plant A. The raw materials were frozen meat products bought from trade companies. Intermediate products generated during the processing stage included pickled, boiled, and cooled products. The processed environmental and facilities samples were collected from associated surfaces using premoistened swabs after cleaning and sanitation procedures were completed. The end products were RTE meat products, prepared for retail and consumption. Samples of these products were delivered to the laboratories within 2 h in a cold chain.

**TABLE 1:** Occurrence of *L. monocytogenes* in the different areas, sources investigated in two RTE meat plants in this study.

**Sampling area**	**Name of samples**	**Plant A**		**Plant B**	
		**Numbers of samples**	**No. pos (%)**	**Numbers of samples**	**No. pos (%)**
Thawing room	Raw materials	7	0 (0)^a^	27	5 (18.5)^a,c^
	Process water	2	0 (0)	4	0 (0)
Trimming room	Raw materials	9	0 (0)^a^	8	1 (12.5)^a^
	Process water	2	0 (0)	0	0 (0)
Pickling room	Intermediate products	12	2 (16.7)^a^	14	1 (7.14)^a^
	Accessory materials	4	2 (50)^a^	3	0 (0)^a^
	Process water	2	0 (0)	2	0 (0)
Boiling room	Intermediate products	14	0 (0)	3	1 (33.3)^a^
	Processing environments and facilities	25	1 (4)	9	0 (0)^a^
Cooled room	Intermediate products	6	2 (16.7)	0	0 (0)^a^
	Processing environments	2	1 (25%)	0	0 (0)^a^
Packaging room	Products	6	1 (16.7)	3	0 (0)^a^
	Processing environments and facilities	17	12 (76.5)^b,d^	46	0 (0)^a^
Sterilizing room	End products	3	0 (0)	9	0 (0)^a^
Total		111	21 (18.9)	128	8 (6.25)^a^

*Number of isolates in rows bearing different letters are significantly different (P < 0.05) between plants A and B.*

*^*a*^Significant difference (P < 0.05) between the two RTE meats plants.*

*^*b*^Significant difference (P < 0.05) in the similar area between the two plants.*

*^*c*^Significant difference (P < 0.05) between thawing room and other areas in plant B.*

*^*d*^Significant difference (P < 0.05) between packaging room and other areas in plant A.*

### Detection of *L. monocytogenes*

*L. monocytogenes* was detected according to the Chinese food safety national standard GB4789.30 (2016). Regarding this standard, environmental swabs and raw and accessory materials were placed into *L. monocytogenes* broth 1 (LB1) for pre-enrichment at 30°C. Afterward, 100 μl of LB1 was placed into *L. monocytogenes* broth 2 (LB2) at 30°C for 24 h. One inoculation loop of LB2 was streaked on polymyxin acriflavine LiCl ceftazidime esculin mannitol agar plate. The isolates were identified as *L. monocytogenes* using standard biochemical tests (catalase, fermentation of dextrose, and Gram staining). The positive control strain used in this study was *L. monocytogenes* ATCC19114.

### PFGE

PFGE for *L. monocytogenes* was performed using the PulseNet International protocol (CDC, 2016). Based on this protocol, *L. monocytogenes* isolates were embedded into agarose plugs. Then, slices of the agarose plugs were digested using *Asc*I (Takara, Dalian, China) for 3 h at 37°C. *Xba*I-digested *Salmonella* Braenderup H9812 DNA was used as a molecular size marker, and electrophoresis was conducted using the CHEF-DRII apparatus (Bio-Rad Laboratories, Hercules, CA, United States).

The running parameters were as follows: 6 V/cm; angle, 120° temperature, 14°C; initial switch, 4 s; final switch, 40 s; and length, 19 h. After electrophoresis, the gel was stained with 50 μl of ethidium bromide (Sigma-Aldrich, Saint Louis, MO) solution (10 mg/ml) in 500 ml of distilled water for 20 min in a covered container, and destained in 500 ml of fresh water for 30 min. Images were captured using the Gel Doc 2000 system (Bio-Rad), converted to TIFF files, and then analyzed using BioNumerics software v 7.6 (Applied Maths, Kortrijk, Belgium). Finally, clustering was performed using the unweighted pair group method with arithmetic mean. The isolates with two or three different band patterns showing a PFGE similarity level ≥ 90% were assigned to the same pulsotype (PT) (Tenover et al., 1995).

### Genomic DNA Extraction and WGS

Overnight cultures of *L. monocytogenes* strains were harvested. Genomic DNA was extracted using the DNeasy Blood & Tissue Kit (QIAGEN, Hilden, Germany) according to the protocol of the manufacturer except that cells were prelysed with lysozyme for 30 min at 37°C, and the proteinase K treatment was extended to 30 min. DNA concentration, quality, and integrity were assessed using a Qubit Fluorometer (Invitrogen, Waltham, MA, United States) and a NanoDrop Spectrophotometer (Thermo Fisher Scientific, Waltham, MA, United States). Sequencing libraries were generated using the TruSeq DNA Sample Preparation Kit (Illumina, San Diego, CA, United States). Then, genome sequencing was performed using the Illumina Hiseq platform (Illumina). Finally, the reads were trimmed and assembled using the CLC Genomics Workbench v7.0 (CLC Bio, Aarhus, Denmark), and the assembled contigs were exported as raw sequencing reads.

These reads were quality checked using FastQC version (v) 0.11.2 and trimmed using Trimmomatic v 0.36. Subsequently, the trimmed reads were assembled using BioNumerics v 7.6, and the assembled sequence was used for further analysis.

A total of 23 *L. monocytogenes* isolates including 16 ST5 isolates and 3 ST121 isolates in this study, and 4 other ST5 isolates from food and patients as outgroup reference were analyzed using WGS.

### Serogroup, MLST, and Pathogenic Island Determination

Four major serogroups (IIa: serotypes 1/2a, 3a, and 3c; IIb: 1/2b, 3b, and 7; IIc: 1/2c and 3c; and IVb: 4b, 4d, and 4e) could be identified using specific genes: *lmo*0737, *lmo*1118, *ORF2819*, *ORF2110*, and *prs* (Burall et al., 2015). The sequence data for these five genes were extracted from the genome data. Serogroups were determined by the presence or absence of the five genes using BLAST.

STs were assigned using BioNumerics v 7.6 (Applied Maths, Kortrijk, Belgium) according to the classical seven housekeeping loci MLST scheme (Ragon et al., 2008), and sequence data of the isolates were extracted from their genome data.

Virulence associated genes extracted from WGS data using the BioNumerics software were added to the Virulence Factor Database (MOH Key Laboratory of Systems Biology of Pathogen, Institute of Pathogen Biology, Beijing, China)^1^ to identify *Listeria* pathogenicity island-1, island-2, island-3, and island-4 (shown as LIPI-1 to LIPI-4).

### cgMLST Characterization

cgMLST typing was conducted based on the profile of 1,748 loci in the BIGSdb Pasteur cgMLST.^2^ The WGS tools of BioNumerics v 7.6 (Applied Maths) was used for analysis with the integrated 1,748 loci cgMLST scheme (Moura et al., 2016). Cluster analysis was conducted by applying a complete linkage using the BioNumerics software. *L. monocytogenes* isolates showing ≤ 7 allelic differences and belonging to the same cgMLST type were considered potentially epidemiologically linked (Moura et al., 2016).

### SNP Analysis

SNP analysis was conducted using BioNumerics. Strict filtering of SNPs at software settings was applied with the reference genome of LM19057. Considering the possible evolution over time of a population of persistent strains in its environment, a relaxed 25-SNP threshold was applied to define strains as belonging to the same cluster (Gerner-Smidt et al., 2019; Fagerlund et al., 2020).

### Statistical Analysis

The contamination level of *L. monocytogenes* isolates between plant A and plant B, and in different areas in the same plants, was calculated using prevalence ratios and *p*-value. The analysis was performed using the chi-square test, where *p <* 0.05 was considered statistically significant.

## Results

### Isolation and Identification of *L. monocytogenes*

A total of 29 of the overall 239 samples (12.1%) were positive for *L. monocytogenes*, with 21 (18.9%) and 8 (6.25%) samples isolated from plants A and B, respectively. For both plants, low contamination rates were reported in five areas, including trimming, pickling, boiling, cooled, and sterilizing rooms (*p* > 0.05). However, the packaging room at plant A was the most contaminated area (14, 56%; *p <* 0.05), with a maximum level of 76.5% for the processing environments (*p <* 0.05). For other areas, no obvious differences in *L. monocytogenes* occurrence between the two plants were found in this study. At plant B, *L. monocytogenes* was isolated from the thawing, trimming, pickling, and boiling rooms. However, the thawing room had the highest contaminated rates with a peak of 18.5% for raw materials (*p <* 0.05). At plant A, *L. monocytogenes* was isolated from pickling, cooling, and packaging rooms; the packaging room was the most contaminated, with the maximum level (76.5%) for processing environments (*p <* 0.05). More materials regarding the occurrence and distribution of *L. monocytogenes* at the two plants and their statistical comparisons are presented in Table 1.

### Molecular Characteristics of *L. monocytogenes*

**FIGURE 1:**
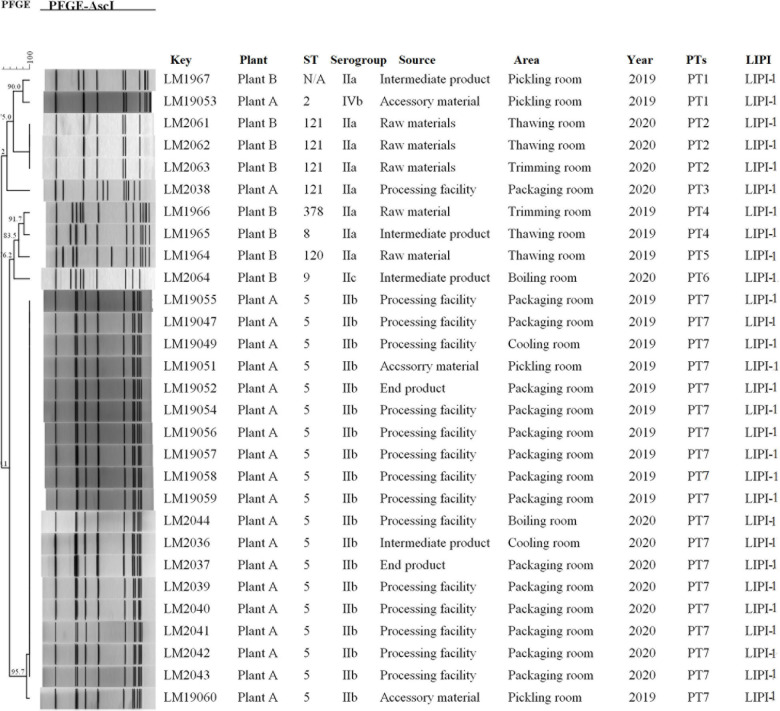
Relationships among the *L. monocytogenes* isolates between of PFGE. The 29 *L. monocytogenes* isolates were analyzed by PFGE using *Asc*I. The corresponding data, including the name of the isolate (Key), name of the plant (Plant), MLST type (ST), serogroup, source, area, separation time (Year), PFGE type, and pathogenic island (LIPI) type, are shown along with the dendrogram to the right.

Molecular typing using WGS data showed that 29 *L. monocytogenes* isolates were typed into nine STs, and all 29 isolates were grouped into four serogroups. A total of 19 out of 29 isolates belonged to ST5 (IIb), followed by four to ST121 (IIa) and one each to ST120 (IIa), ST2 (IVb), ST9 (IIc), ST378 (IIa), and ST8 (IIa). One *L. monocytogenes* isolate was identified as a new ST. All of the isolates carried LIPI-1. All 19 *L. monocytogenes* isolates (19/21) of ST5 were from plant A (11 from 2019 and 8 from 2020). Furthermore, 14 of the 19 isolates with ST5 were from processing environment and facility samples, 2 from end products, 2 from accessory materials, and 1 from intermediate products. Three of four isolates with ST121 were from plant B in 2020. All three *L. monocytogenes* isolates were from raw materials (Figure 1).

PFGE analysis typed the 29 isolates into seven pulsotypes (PT1–7) (Figure 1). *L. monocytogenes* isolates in PT7 and PT2 had indistinguishable PFGE profiles (> 95.7%) and accounted for 75.9% (22/29) of these isolates. *L. monocytogenes* isolates in PT7 were ST5 (IIb), which were isolated during 2019–2020 in plant A. However, *L. monocytogenes* isolates in PT2 were ST121, which were isolated in 2020 at plant B. Two PTs (PT1 and PT4) were demonstrated in two isolates. The other two PTs (PT3 and PT6) were presented by only a single isolate each.

### cgMLST

**FIGURE 2:**
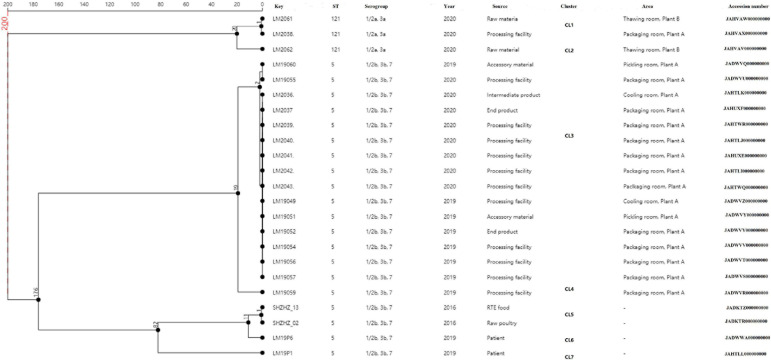
Minimum spanning tree of cgMLST data for the 23 *L. monocytogenes* isolates. The multiplication by 1 in the tree represents the number of different alleles among isolates. The corresponding data, including the name of the isolates (Key), ST, serogroup, year, source, cluster, area, and accession number, are shown along with the dendrogram to the right.

A total of 23 *L. monocytogenes* ST5 and ST121 isolates were used for cgMLST analysis (Figure 2). All the ST5 isolates were from plant A, with 12 out of the 16 isolates from packaging room during 2019–2020, including 10 isolates from the processing facilities and 2 isolates from end products. Two ST5 isolates were from the cooling room (processing facility and intermediate product), whereas another two ST5 isolates were from accessory products in the pickling room. Two *L. monocytogenes* isolates from food and two isolates from patients were used as outgroup reference isolates. These isolates were compared using cgMLST. Seven clusters (CL1–CL7) were obtained as shown in Figure 2. Of 16 ST5 isolates from plant A, 15 belonged to CL3 and had up to two alleles based on cgMLST. A total of 176 alleles were found between the isolates in CL3 and other CLs (CL5–CL7). More than 200 alleles were found between ST5 isolates and ST121 isolates.

### SNPs

**FIGURE 3:**
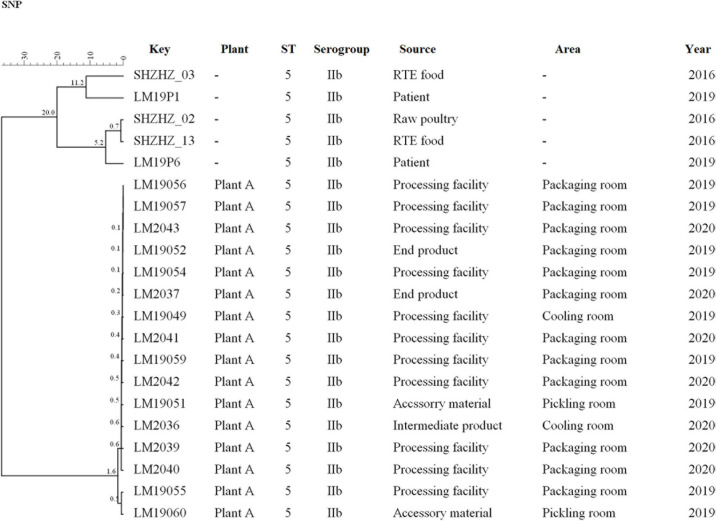
Clustering of the 16 *L. monocytogenes* based on SNPs by BioNumerics software using five unrelated *L. monocytogenes* isolates for comparison. LM19057 isolated from plant A was used as reference. The corresponding data, including the name of these isolates (Key), source (Plant), ST, serogroup, and year, are shown along with the dendrogram to the right.

The same ST5 isolates were used for SNPs analysis (Figure 3). Core SNP analysis was performed to deepen the genetic relationship among these ST5 isolates. The obtained results, according to the 25-SNP threshold, confirmed what was observed from the cgMLST results, identifying the same clusters with the same strain composition (Figure 3). According to the SNP matrix, in cluster 5, the number of SNPs in strains ranged from 0 to 21. The seven outlier strains differed in the number of SNPs from 7 to 113 and differed by 200 SNPs from strains of cluster 5.

## Discussion

The control of *L. monocytogenes* in RTE meat processing plants is an ongoing and important challenge. The introduction of *L. monocytogenes* to processing plants continues to occur, and persistent *L. monocytogenes* isolates can exist in food processing plants because of their strong ability to survive in different types of conditions (Conficoni et al., 2016). Therefore, samples from different areas in the two RTE meat processing plants were collected to investigate the distribution of *L. monocytogenes* and track the sources of contamination. A total of 239 samples were collected in the two RTE meat processing plants during 2019–2020 in Shanghai (Table 1); 58 of these samples were from raw and accessory materials, and 12 samples were from process water, by which *L. monocytogenes* isolates could enter food processing environments (Marriott et al., 2018). Overall, 99 of the 239 samples were from processing environments and facilities, such as floor, wall, transport units, pipes, trash can, and weighing tools, as well as hands, clothes, and shoes of the handlers. These niches can harbor sites where *L. monocytogenes* can survive and even grow (Buchanan et al., 2017). Overall, 70 of the 239 samples were intermediate and end products, collected and also identified to be contaminated by *L. monocytogenes*.

Our study confirmed the different contamination models of *L. monocytogenes* observed in the two RTE meat plants. Indeed, a higher level of *L. monocytogenes* contamination occurred in the packaging room than in other areas (*p <* 0.05), indicating this may be the high-risk area. At the same plant, the positive rate of *L. monocytogenes* from samples in the packaging room was significantly higher than that in other rooms (*p <* 0.05). These results suggest that contamination of *L. monocytogenes* in the packaging room was more likely to occur. In packaging rooms, more of the tasks required human handling, such as weighing, cutting, and packaging. Furthermore, *L. monocytogenes* isolates in CL5 were found in the clothes and shoes of the workers, as well as facilities and environments in packaging rooms; this suggests the possibility of cross contamination. Accordingly, on the one hand, it is essential to pay attention to the hygiene of the workers, i.e., their clothes needed to be cleaned and disinfected regularly. On the other hand, it is vital to clean and disinfect the facilities and environments regularly. Moreover, *L. monocytogenes* isolates have been detected in 2019 as well as in 2020. Therefore, regular cleaning and disinfection are essential.

Six *L. monocytogenes* isolates were detected in raw materials at plant B. However, *L. monocytogenes* was not detected in processing environments and facilities at plant B. These results suggested that raw materials contaminated with *L. monocytogenes* were the means whereby bacterium could enter food processing environments. However, this contamination could be eradicated by some measure. Raw materials should be cleaned and disinfected, and good hygiene of the plants should be essential.

The PFGE profiles of 19 *L. monocytogenes* isolates in PT7 were indistinguishable (> 95.7%), suggesting that they were from the same ancestor. The epidemiological data showed that these 19 *L. monocytogenes* isolates detected at plant A during 2019–2020 were persistent isolates that might have been presented since 2 years at the plant. A similar contamination model reported that when *L. monocytogenes* isolates entered food processing plants, recontamination and persistence frequently occurred (Chambel et al., 2007), and such samples represented 65.5% (19/29) of the overall *L. monocytogenes* isolates. The presence of *L. monocytogenes* in PT7 in the cooling, pickling, boiling, and packaging rooms and their circulation at plant A through the transmission between food products and the facilities, probably represented the mechanism whereby *L. monocytogenes* could persist in the food processing plants. Indeed, a significant difference (*p <* 0.05) in the occurrence of contamination between plants A and B was observed in the packaging room, and a significant difference (*p <* 0.05) was also observed between the packaging room and other areas at plant A. This contamination model suggested the existence of cross contamination between different areas within the same plant and reflected the operative features of a plant regarding sanitization procedures and behavior of workers. Based on the findings observed in the packaging room at plant A, the hands, clothes, and shoes of the handlers were contaminated with *L. monocytogenes* isolates belonging to PT7 and could be considered carriers for processing environments, facilities, and even end products. In contrast, processing facilities, such as conveyors, and the inside and outside surface of facilities were significant niches that supported the tendency of *L. monocytogenes* to persist in processing facilities because of the difficulty in completely eradicating the bacterium from these areas. It has been reported that similar hard-to-clean surfaces allow the bacteria to survive or even proliferate, thereby making it difficult to completely eradicate from these surfaces (Ferreira et al., 2014). Therefore, ensuring correct sanitization practices is essential to avoid cross contamination to products. Where necessary, facilities should be autoclaved to completely eradicate *L. monocytogenes.*

There was a high diversity of PFGE patterns of *L. monocytogenes* from plant B. Eight isolates were typed into five PTs (PT1–PT2 and PT4–PT6). ST121-IIa was predominant with three *L. monocytogenes* isolates identified from raw materials in plant B in 2020. However, one ST121-IIa was detected in the processing facility at plant A in 2020, which belonged to PT3. The similarity of the isolate in PT3 to ST121 isolates in PT2 was low. Furthermore, only one ST121 isolate was detected in plant A. It was difficult to judge how ST121 isolates entered plant A. Although there was no relationship between plant A and plant B, these results could suggest that ST121 *L. monocytogenes* could enter plants via raw materials. Raw meat has rarely been implicated in foodborne disease, although this may be considered a potential source of domestic cross contamination of other foods (Thévenot et al., 2006). ST121 *L. monocytogenes* has been reported to persist in food processing facilities (Naditz et al., 2019). Therefore, strengthening surveillance of *L. monocytogenes* in raw materials as well as food processing environments is essential to prevent contamination of *L. monocytogenes*.

In this study, WGS was used to obtain more detailed information on the genetic similarity between isolates and has clear advantages over PFGE (Zhen et al., 2017). The indistinguishable *L. monocytogenes* isolates that have the same PFGE pattern could be differentiated by cgMLST (Figure 2). cgMLST analysis of *L. monocytogenes* isolates in PT7 exhibited up to nine allelic differences, and these isolates could be identified as being the same clone. The closest pairwise differences between these ST5 isolates from plant A ranged from 0 to 16 SNPs. These numbers were well below the threshold of 25 SNPs commonly employed during outbreak investigations as an indication that two isolates had originated in the same facility (Wang et al., 2018; Allard et al., 2019). This indicated that persistence of *L. monocytogenes* at plant A was the most common scenario. These isolates were from different areas, such as the packaging, cooling, and pickling rooms, which suggested that clone transmission had occurred at plant A during 2019–2020 by cross-transmission. This cross contamination has been reported as the primary route of contamination by *L. monocytogenes* in RTE food products in food processing environments (Møretrø and Langsrud, 2004; Ferreira et al., 2014); clones of *L. monocytogenes* are known to have persisted for years in processing environments, including those in meat industries (Cherifi et al., 2018; Melero et al., 2019).

The primary origin of ST5 from plant A is unknown. In-depth epidemiological studies have identified that ST5 isolates can be highly abundant in foods and in food processing environments (Muhterem-Uyar et al., 2018; Naditz et al., 2019). Our previous study indicated that the predominant *L. monocytogenes* isolates from both food and clinical patients in Shanghai were ST5 (Zhang et al., 2020). Why do ST5 isolates tend to be persistent in food processing environments? At the genome level, the ST5 isolates contain plasmids harboring an efflux pump system (*bcrABC* cassette) and heavy metal resistance genes, which may be important for the persistence of ST5 isolates in food processing environments (Muhterem-Uyar et al., 2018). In contrast, our previous study proved that these ST5 isolates at plant A could form biofilms, which provided a protective environment for bacterial survival and thus increased the risk of subsequent contamination (Colagiorgi et al., 2017; Zhang et al., 2021). Although ST5 isolates with LIPI-1 were not hypervirulent because they lack LIPI-3 or LIPI-4 (Manso et al., 2019), they still have caused many sporadic patients (Zhang et al., 2020). Several outbreaks caused by *L. monocytogenes* have been linked to ST5 isolates (Buchanan et al., 2017). Therefore, the potential risk of pathogenicity is worth noting, and further studies are needed to uncover the trait that enables persistence of ST5 in food processing environments.

Raw meat is a common source of introduction of *L. monocytogenes* into food processing environments (Fagerlund et al., 2020). In this study, raw materials were also positive for *L. monocytogenes* isolates. Raw meat distribution chains in China are complex, and there are many different raw meat suppliers who sold raw meat to different meat processing plants. This may explain why the positive rate of *L. monocytogenes* occurrence was different between plants A and B. Furthermore, a total of three STs (ST378, ST121, and ST120) from raw materials were detected in plant B. However, these STs were only detected once, suggesting that these were transient isolates. However, transient isolates could change to persistent isolates under specific conditions (Naditz et al., 2019), and detection of *L. monocytogenes* from raw materials should be prioritized to ensure food safety. Furthermore, detection of *L. monocytogenes* isolates from upstream in the raw material chains was required to potentially identify the primary sources of *L. monocytogenes* isolates.

## Conclusion

Our study demonstrated a two-contamination model of *L. monocytogenes* isolates at different RTE meat processing plants in Shanghai. Using PFGE, ST5 isolates at plant A were confirmed as persistent isolates and may have been persisted here for an extended period (at least 2 years). Clone transmission was confirmed using WGS data from processing environments and facilities by cross contamination. At plant B, *L. monocytogenes* isolates could enter the food processing environment from raw materials. However, these isolates were considered as transient isolates by PFGE and were not detected in the processing environments, facilities, and RTE products. These results suggested that continuous monitoring, stringent surveillance, and source tracking are crucial to guarantee food safety.

## Data Availability Statement

The datasets presented in this study can be found in online repositories. The names of the repository/repositories and accession number(s) can be found in the article/supplementary material.

## Author Contributions

HZ and JW collected samples, analyzed samples, performed WGS, and analyzed the data. HZ drafted the manuscript. XZ designed the study and revised this manuscript. ZC and XL tested the samples. YY and JW performed the PFGE. YLY and XW were involved in the collection of isolates. QD and XZ revised this manuscript. All authors contributed to the article and approved the submitted version.

## Conflict of Interest

The authors declare that the research was conducted in the absence of any commercial or financial relationships that could be construed as a potential conflict of interest.

## Publisher’s Note

All claims expressed in this article are solely those of the authors and do not necessarily represent those of their affiliated organizations, or those of the publisher, the editors and the reviewers. Any product that may be evaluated in this article, or claim that may be made by its manufacturer, is not guaranteed or endorsed by the publisher.

## Footnotes

1
http://www.mgc.ac.cn

2
http://bigsdb.pasteur.fr/listeria/listeria.html
